# Hydrogen therapy may be an effective and specific novel treatment for Acute Graft-versus-host disease (GVHD)

**DOI:** 10.1111/jcmm.12081

**Published:** 2013-06-07

**Authors:** Liren Qian, Jianliang Shen

**Affiliations:** a Department of Haematology, Naval General HospitalBeijing, China

**Keywords:** Hydrogen, graft-versus-host disease, antioxidant, cytokine

## Abstract

Allogeneic haematopoietic stem cell transplantation (HSCT) has been widely used for the treatment of haematological malignant and non-malignant haematologic diseases. However, acute graft-versus-host disease (aGVHD) is a kind of severe complication of HSCT limiting its application. Cytokines such as tumour necrosis factor-α (TNF-α), IL-6 play an extremely important role in the formation and development of aGVHD. Besides, the oxidation phenomena and/or the formation of free radicals have been suggested to be causally related to various haematological disorders including aGVHD. Reactive oxygen species (ROS), such as hydroxyl radicals, play an important role in the formation and development of aGVHD. Hydrogen has been reported to have the ability to inhibit levels of cytokines such as TNF, IL-6 *in vivo*. Our recent studies provided evidence that hydrogen inhalation can selectively reduce cytotoxic oxygen radicals and exert antioxidant effects. Therefore, we suggested that hydrogen may have therapeutic effects on aGVHD. This hypothesis entails many experimentally testable predictions. We propose the experimental study by detecting complete blood counts (CBC) and Clinic signs of aGVHD mice. We also propose to detect the levels of TNF-α, IL-2, IL-1β, IL-6 which play important roles in the pathogenesis of aGVHD. To discover potential mechanisms of the therapeutic effects of hydrogen on the aGVHD model, we will examine gene-expression profiles. This study will open a new therapeutic avenue combining the field of therapeutic medical gases and aGVHD. This theory is original and probably of importance, because therapeutic medical gases have never been used for aGVHD previously.

## Background

Hydrogen is the most abundant chemical element. Hydrogen gas has been extensively used in chemical field such as fuel processing, fertilizer production (3H2 + N2 → 2NH3) and so on. It is a colourless, odourless, non-metallic, tasteless, highly flammable diatomic gas which was considered as a physiological inert gas.

Early in 1975, Dole *et al*. [1]found that inhalation of a mixture (2.5% O_2_ and 97.5% H_2_) at a total of 0.8 MPa for 2 weeks caused a marked regression of the skin squamous cell carcinoma in a mouse model, and they tried to elucidate the phenomenon with the possibility of H_2_ as a free radical decay catalyser. In 1988, A paper by Buxton *et al*. [2] demonstrated that H_2_ could reduce hydroxyl radicals (·OH) produced by radiolysis or photolysis of H_2_O in cell-free systems. In 2001, it was demonstrated that schistosomiasis-associated chronic liver inflammation was significantly attenuated by one normal atmosphere supplemented with 0.7 MPa H_2_ in mice [3]. However, these investigations did not draw attention from researchers.

In 2007, Ohsawa *et al*. [4]discovered that hydrogen gas has antioxidant and antiapoptotic properties that protect the brain against ischaemia–reperfusion injury and stroke by selectively neutralizing hydroxyl and peroxynitrite radicals. Since then, hydrogen gas has come to the forefront of therapeutic medical gas research. Recent basic and clinical research has revealed that hydrogen is an important physiological regulatory factor with antioxidant, anti-inflammatory and antiapoptotic protective effects on cells and organs [5–10], proving that hydrogen could down-regulate cytokines, including CCL2, IL-1β, IL-6, IL-12, TNF-α, etc. We also have proposed and proved that hydrogen has radioprotective effects in cultured cells and mice [11–17]. It also has been demonstrated that H_2_ is effective in the prevention of cerebral, myocardial, hepatic ischaemia–reperfusion injuries and other injuries [18, 19].

Since 2009, hydrogen was applied on the field of organ transplantation including intestinal transplantation, lung transplantation, renal transplantation and heart transplantation. It was demonstrated that hydrogen could protect allograft function in those models [19–23]. However, the potential effect of hydrogen gas on another transplantation type is largely ignored. That type is the allogeneic haematopoietic stem cell transplantation.

## Presentation of the hypothesis

Allogeneic HSCT is a potentially curative therapy for many malignant haematologic disorders, while it is restricted by its severe complications. One major complication associated with HSCT is aGVHD, which is a major cause of death following allo-HSCT. Three complex stages are involved in the pathophysiology of aGVHD [24]. Stage I involves tissue damage and cellular activation induced by preconditioning. Stage II involves activation of donor lymphocytes (T cells). In Stage III, cellular and inflammatory factors are released, including TNF-α, interleukin (IL)-1 and IL-6 *et al*. These cytotoxic molecules directly attack various host tissues and underlie the clinical manifestations of aGVHD [25, 26]. The activated cells also produce a large number of harmful free radicals resulting in severe cell damage, which also play an important role in the development of aGVHD [27].

Nowadays, the standard initial therapy for aGVHD includes the use of high-dose steroids, which results in about 40% complete response (CR) rate [28]. This CR rate is unsatisfactory, and in patients who have less than a CR, there is high mortality, both from the aGVHD itself and from the steroid-related infectious complications. Treatment of steroid-resistant aGVHD is very difficult, with many institutions using monoclonal antibody treating steroid-resistant aGVHD patients, including anti-TNF-α monoclonal antibody(mAb), anti-CD52 mAb, anti-CDl47 mAb (ABX.CBL), anti-CD3 mAb, *et al*. But their therapeutic effects are not ideal, while with high infection rate. Overall, those treatments of aGVHD did not achieve significant breakthroughs. Various researchers have engaged in identifying novel, non-toxic, effective and convenient drugs to cure or alleviate aGVHD.

**Fig. 1:**
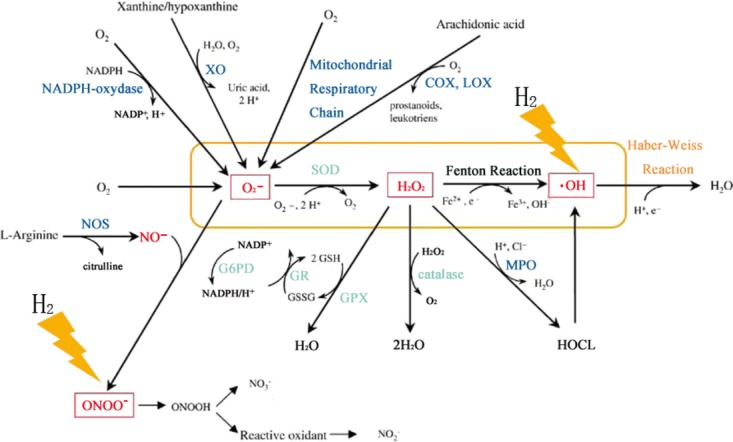
Cytotoxic oxygen radicals that hydrogen could selectively reduce.

Our hypothesis is that hydrogen gas may have therapeutic effects on aGVHD. Our hypothesis is based on the theory that hydrogen could down-regulate cytokines, CCL2, IL-1β, IL-6, IL-12, TNF-α, *et al*. and selectively reduce hydroxyl and peroxynitrite radicals (Fig. 1). In addition, H_2_ has other advantages; it has demonstrated that H_2_ has non-toxicity at any pressure [29]. It can penetrate biomembranes and diffuse into the cytosol, mitochondria and nucleus to protect nuclear DNA and mitochondria [30].

As those cytokines including IL-6, IL-1, TNF-α, *et al*. and free radicals have been suggested to play great important roles in the formation and development of aGVHD as discussed above, we suggested that hydrogen gas may be potentially effective for aGVHD by down-regulating cytokines and selectively reducing hydroxyl and peroxynitrite radicals.

## Testing the hypothesis

**Fig. 2:**
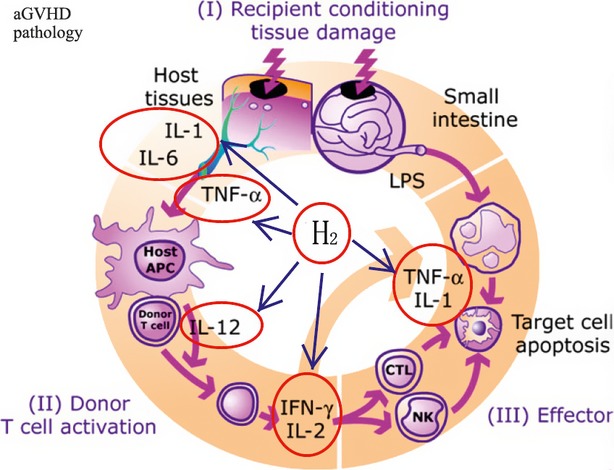
Cytokines that hydrogen could down-regulate (→) in the pathogenesis of aGVHD. Adapted from Ferrara [38].

For testing the hypothesis, hydrogen gas could be administered by two ways: First, it may be administered to patients *via* inhalation as room air at safe concentrations (<4.6% in air by volume). Second, we can dissolve hydrogen gas into water delivering it as drinking water. This may be more practical in daily life and more suitable for daily consumption for therapeutic use. Hydrogen-rich drinking water can be generated by several methods including dissolving electrolysed hydrogen into pure water, dissolving hydrogen into water under high pressure and utilizing electrochemical reaction of magnesium with water. We propose the experimental study by detecting CBC, Clinic signs of aGVHD mice (weight loss, posture, activity, fur change, skin integrity) as described previously [31, 32]. We also propose to detect the levels of TNF-α, IL-2, IL-1β, IL-6 as described previously [33, 34], which have been demonstrated to play important roles in the pathogenesis of aGVHD. And plasma malondialdehyde (MDA), 8-hydroxydeoxyguanosine (8-OHdG) and endogenous antioxidants such as SOD, GSH will also be detected *in vivo*. To discover potential mechanisms of the therapeutic effects of hydrogen on the aGVHD model, we will examine gene-expression profiles, such as expression of Bcl-2, Bax, Caspase-3, Caspase-8 [35, 36]. On the basis of these experimental designs, we preliminarily demonstrated the therapeutic effects of hydrogen on aGVHD in a mice model [37]. We demonstrated that hydrogen treatment could protect mice from lethal GVHD and improve clinic syndrome of aGVHD mice and also could promote the recovery of white blood cells of aGVHD mice. We also examined serum cytokine levels. Cytokines, such as TNF-α, IL-2 were also reduced by hydrogen, which play critical roles in the development of aGVHD (Fig. 2).

## Implications of the hypothesis

This study will open a new therapeutic avenue combining the field of therapeutic medical gases and aGVHD. This theory is original and probably of importance, because therapeutic medical gases has never been used for aGVHD previously. We believe that *in vitro* and *in vivo* work for hydrogen gas on aGVHD would come in great numbers as soon as possible. In view of the high lethality rate of aGVHD, hydrogen gas may give us more hope for greater survival with few side effects.
